# Eye to eye with *Thelazia*-infected canids in Central European forests

**DOI:** 10.1016/j.crpvbd.2026.100353

**Published:** 2026-01-21

**Authors:** Eszter Nagy, Rebeka Ráhel Nagy, Máté Miklós, Sándor Szekeres, Bawan Mustafa Abdalrahman, Gábor Földvári, Lajos Rózsa, Éva Fok, Tamás Sréter, Tamás Tari, Melinda Kovács, Ágnes Csivincsik, Gábor Nagy

**Affiliations:** a Institute of Wildlife Management and Wildlife Biology, Faculty of Forestry, University of Sopron, Sopron, Hungary; b Institute of Evolution, HUN-REN Centre for Ecological Research, Budapest, Hungary; c Centre for Eco-Epidemiology, National Laboratory for Health Security, Budapest, Hungary; d Department of Parasitology and Zoology, University of Veterinary Medicine, Budapest, Hungary; e HUN-REN-UVMB Climate Change: New Blood-Sucking Parasites and Vector-Borne Pathogens Research Group, Budapest, Hungary; f One Health Working Group, Kaposvár Campus, Hungarian University of Agriculture and Life Sciences, Kaposvár, Hungary; g National Center for Public Health and Pharmacy, Laboratory Department of Bacteriology, Mycology and Parasitology, Budapest, Hungary

**Keywords:** *Thelazia callipaeda*, European beech, Golden jackal, Red fox, Summer heat-moisture index

## Abstract

The oriental eyeworm *Thelazia callipaeda* has been present in Europe since the late 1980s. Its occurrence in the Carpathian Basin has been known since 2014. Despite the central position of Hungary in the radial expansion of *T. callipaeda* in Central and Eastern Europe, no comprehensive surveillance has been conducted to date to determine the reservoir role of wild carnivores within the Carpathian Basin. The study involved the analysis of samples from 180 red foxes (*Vulpes vulpes*), 119 European badgers (*Meles meles*), 62 golden jackals (*Canis aureus*), and 10 stone martens (*Martes foina*) harvested in the framework of an authorised wildlife management programme. Among the mustelids (family Mustelidae), no infected individuals were found. In the red fox, prevalence and mean intensity were 12.2% (95% CI: 8.0–18.0%) and 2.6 (95% CI: 1.7–4.9), respectively; while in the golden jackal, these values were 9.7% (95% CI: 4.3–20.0%) and 3.0 (95% CI: 1.5–6.5), respectively. The difference in prevalence and mean intensity of infection between the two hosts proved non-significant. The generalised linear models suggested that the presence of hygrophilous beech (*Fagus sylvatica*) forests positively influenced the occurrence of infection in wild carnivores. Although the receiver operating characteristic (ROC) curve analysis showed only a modest discriminatory power for the models, these findings highlighted the potential of humidity in the spread of *T. callipaeda* in the Carpathian Basin.

## Highlights

• *Thelazia callipaeda* was recorded in wild carnivores in southwestern Hungary.
• Infection was confirmed in red foxes (*Vulpes vulpes*) and golden jackals (*Canis aureus*).
• All examined mustelids (family Mustelidae) tested negative for the parasite.
• Prevalence values were comparable to those reported in neighbouring Croatia.
• A possible association was observed between beech forests and infection risk.

## Graphical abstract

**Figure:**
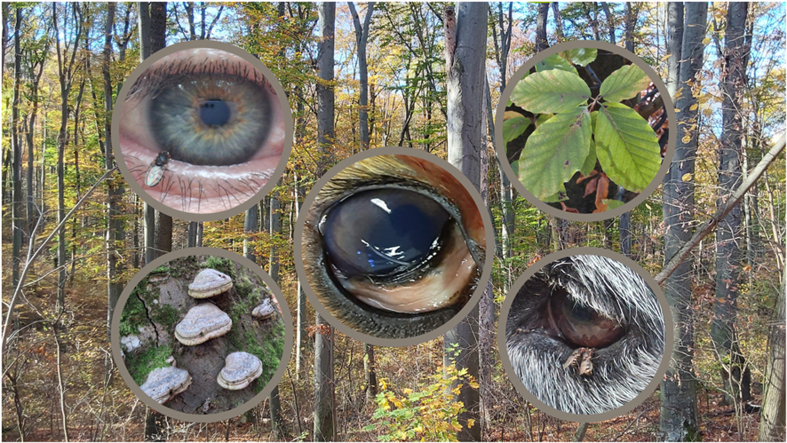

## 1 Introduction

The oriental eyeworm (*Thelazia callipaeda*) is a zoonotic parasite that lives in the orbital cavity of its definitive hosts, mostly canids (Otranto et al., 2003a). However, wild rabbits (*Oryctolagus cuniculus*), brown hares (*Lepus europaeus*), brown bears (*Ursus arctos*) (do Vale et al., 2020), and mustelids (Odoevskaya et al., 2015; Ionică et al., 2019; Gagović et al., 2025) have also been shown to act as definitive hosts. The intermediate hosts that provide a habitat for larval development belong to the family Drosophilidae. The lachryphagous behaviour of the intermediate host ensures the transmission of the infection (Otranto et al., 2006; do Vale et al., 2020). As a vector-borne parasite, *T. callipaeda* is considered sensitive to climatic conditions. Based on this hypothesis, its distribution area is predicted to expand as a result of climate change (Palfreyman et al., 2018). Moreover, the spread of *T. callipaeda* in Europe is a prime example of biological invasion. Since its first detection in southern Europe in 1989, it has been spreading continuously towards northern territories (Caminade et al., 2019; do Vale et al., 2020; Bertos et al., 2023). The role of wild canids as both reservoirs and spreaders of *T. callipaeda* has been highlighted several times (Otranto et al., 2007; Hodžić et al., 2014; Bezerra-Santos et al., 2022; Gagović et al., 2025). Furthermore, this parasite also poses a health risk to both endangered species (Bertos et al., 2023) and to vulnerable human populations (Otranto et al., 2005a, 2005b; Máca and Otranto, 2014). Consequently, *T. callipaeda* presents a One Health challenge at the synanthropic-sylvatic interface, representing a form of human-wildlife conflict (do Vale et al., 2020; Salerno et al., 2025).

The early history of *T. callipaeda* was summarised by Faust (1928). It was first described as a parasite of dogs in Punjab, India, in 1910. In the same year in Burma (now Myanmar), another dog case was reported. The first human patient was diagnosed with thelaziosis in Beijing in 1917. Due to the seasonality of the parasite’s transmission, the role of a potential vector was suspected (Faust, 1928). Drosophilid flies were identified as vectors of *T. callipaeda* in the early 1960s, in the Russian Far East (Kozlov, 1961). In Europe, the oriental eyeworm was first detected in 1989. During a survey conducted in Piedmont, among 314 dogs, 16 (5.1%) were confirmed to be infected with the parasite (Rossi and Bertaglia, 1989). Some years later, a parallel study that surveyed a temperate zone (Piedmont) and a Mediterranean area (Basilicata region) of Italy, evidenced that the infection occurred in the dog populations of both areas (Otranto et al., 2003a). After the discovery of the Italian focus, the parasite was detected in Switzerland (Malacrida et al., 2008), France (Dorchies et al., 2007), Spain (Miró et al., 2011), and Portugal (Seixas et al., 2018). Meanwhile, the endemic state in the Apennine Peninsula appears to have spread eastward as well, with the first cases reported from Croatia, Bosnia and Herzegovina (Hodžić et al., 2014), and Serbia (Gajić et al., 2014).

Although the first case in Germany was detected in 2004 (Hermosilla et al., 2004), it was ascertained during the investigation of the case that the infected dog frequently stayed in Italy. This finding highlighted the epidemiological risk of animal transports from endemic areas. The same phenomenon was observed on the British Isles. Although researchers from both the UK (Graham-Brown et al., 2017) and Ireland (de Waal et al., 2019) reported imported cases of *T. callipaeda*, this area has remained free of autochthonous cases so far. These events also highlighted the urgency of changing attitudes toward parasitic diseases that can be transported unnoticed from endemic to previously free areas (Wright and Elsheikha, 2017).

The epidemic’s frontier may have arrived in the Carpathian Basin in the early 2010s. The central position and ecological suitability of this geographical region (Otranto et al., 2006) supported the radial expansion of *T. callipaeda*. Research groups from Hungary (Fok et al., 2016), Romania (Mihalca et al., 2015), Slovakia (Čabanová et al., 2018), Greece (Diakou et al., 2015), Moldova (Dumitrache et al., 2019), Poland (Rolbiecki et al., 2021), and Austria (Unterköfler et al., 2023) reported the first cases of the parasite within a few years.

The emergence of the new zoonotic parasite in Europe enhanced the research activities. The extensive spread on the continent, parallel with climate change, highlighted the role of the vector species. It was confirmed that the competent vector in Europe was the variegated fruit fly, *Phortica variegata* (Insecta: Diptera: Drosophilidae) (Otranto et al., 2005a). Besides *P. variegata*, other lachryphagous fruit flies, such as *Phortica semivirgo*, *Phortica erinacea*, *Phortica goetzi*, and *Phortica oldenbergi*, were identified as potential vectors in European ecosystems (Máca and Otranto, 2014; González et al., 2025). Based on the climatic optimum (20–25 °C ambient temperature and 50–75% relative humidity) of *P. variegata* during its breeding season, Otranto et al. (2006) constructed an ecological niche model which predicted that Central Europe was especially suitable for this vector and thus, for the spread of thelaziosis. This model determined the southern part of the British Isles and Scandinavia as the northernmost borders of the expected distribution area.

Since the epidemiological situations vary remarkably within the distribution area, researchers compared climatic and ecological conditions of hyperendemic regions. There is a consensus that humid forest ecosystems (Kozlov, 1963; Otranto et al., 2005a, 2006; Roggero et al., 2010; Hodžić et al., 2014; Máca and Otranto, 2014), and especially the presence of oak (*Quercus* spp.) forests, are important for the maintenance of dense vector populations (Palfreyman et al., 2018; Marino et al., 2020; Pombi et al., 2020; Bernardini et al., 2022, 2024; González et al., 2025). The importance of geomorphological relief was also evidenced as the highest prevalences were detected in endemic regions of mountainous or at least hilly landscapes: 64.5% in Kumamoto, Japan (Otranto et al., 2003a); up to 75.8% in dogs of Khabarovsk Krai district, in the Sikhote-Alin Mountains of the Russian Far East (Kozlov, 1963); 50.0% in eastern Bosnia; 60.1% in Accettura, Italy; and up to 68.0% in La Vera Province, Spain (Miró et al., 2011; Marino et al., 2018).

The genetic difference between *T. callipaeda* populations of remote endemic regions was investigated in the historical focus of China and Japan, and in the newly invaded areas of Europe. It is evidenced that the Asian suprapopulation of the parasite possesses a rich genetic diversity with 20 haplotypes (Zhang et al., 2018), while in Europe, Haplotype 1 is widely distributed (Otranto et al., 2005b; Zhang et al., 2018), and a novel variant haplotype (Haplotype 22) has been detected recently in Italy (Cavallero et al., 2025). The phylogenetic closeness of the Asian Haplotype 4 and the European Haplotype 1 supports the Asian origin of the European lineage. However, the estimated divergence time during the Pleistocene does not provide information on when it was likely introduced into Europe (Zhang et al., 2018).

Within the historical distribution area, in Central China, the increase in the number of cases began during the 1980s. The most affected provinces are Hubei and Shandong, where case accumulation is observed in both humans and dogs (Wang et al., 2014). Researchers agree that, besides climatic and geographical factors, socio-economic deprivation is also an important driver of the maintenance and spread of the parasite in the concerned regions throughout the distribution area (Wang et al., 2014; Colella et al., 2016; Zhao et al., 2017; Liu et al., 2020). In Asia, the first human patient was referred to as a “coolie”, a term that meant a low-income worker in the colonial era (Faust, 1928). In southern Italy, the most affected region, is a remote area, “geographically well-defined but also very difficult to access and very poor in terms of human and social contacts with other settlements or communities” (Otranto et al., 2003a).

Based on the experiences collected in the Eurasian distribution areas of *T. callipaeda*, an eco-epidemiological study was designed to reveal the locally acting drivers of the parasite’s transmission. It was hypothesised that an investigation at the local scale could reveal disease processes that are constrained at higher levels; therefore, remain undetected during large-scale studies (Goodin et al., 2018). The research was conducted in a highly forested area, which provided an opportunity to analyse the potential role of the finer-scale landscape features in the maintenance of *T. callipaeda* infection among wild carnivores. Furthermore, it was hypothesised that, due to the potential parasite-accumulating effect of uncared-for rural dogs, carnivores hunted in synanthropic environments were more likely to carry the parasite.

## 2 Materials and methods

### 2.1 Study area and vegetation

**Fig. 1:**
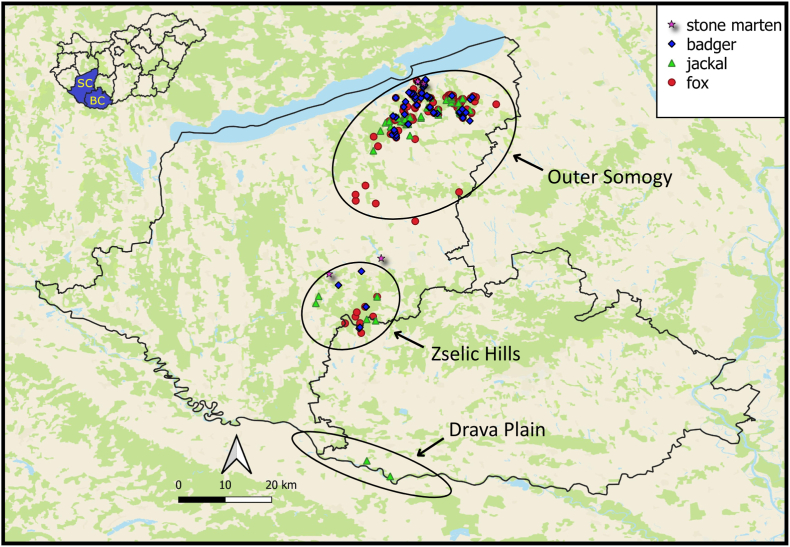
Locations and sampling coordinates of the stone martens, golden jackals, red foxes, and badgers investigated in the study. The animals originated from Somogy (SC) and Baranya counties (BC). *Note*: Black line represents the administrative border of the two counties; the southern border of these counties also marks the national border with Croatia; ellipses indicate the approximate boundary of the sampling areas (Outer Somogy, Zselic Hills, Drava Plain).

The research was conducted in the South Transdanubian region of Hungary (Fig. 1). This area and eastern Croatia adjoin each other along the River Drava, and the two regions share similar biogeographical characteristics. Both possess a diverse landscape mosaic featuring flat plains, rolling hills, and low mountains (Reed et al., 2004). The sub-Mediterranean and sub-Atlantic climates affect both areas, resulting in a transition zone where Western Balkan (Illyrian) plant species coexist with Pannonian flora (Salamon-Albert et al., 2011; Fekete et al., 2014). This region of Hungary is one of the most forested areas supporting dense populations of wild ungulates and the golden jackal (*Canis aureus*) (Kemenszky et al., 2022). Within the study area, a remarkable zonation can be observed in both climate and vegetation from southwest to northeast due to the dual climatic influence. The sampling sites (the Zselic Hills, Outer Somogy, and the Drava Plain) represented different climatic zones of South Transdanubia (Fig. 1). The Drava Plain, being under a strong Mediterranean influence, is warm, and its vegetation is characterised by abundant wet habitats. The Zselic microregion is a hilly area with a cooler and more humid local climate, which results in moisture-indicating forest communities. The northeastern Outer Somogy is the driest zone, with thermophilous oak and steppe oak forests as characteristic plant communities; however, in deep valleys, some isolated spots of hygrophilous vegetation can be observed (Salamon-Albert et al., 2010).

### 2.2 Animals

The study was conducted from November 2024 to July 2025. The sampling period was timed to fall between the 2024 active season and the subsequent activity peak of the intermediate host. All available carcasses legally hunted in the study area, a total of 371 wild carnivore specimens, were collected to determine their *T. callipaeda* infections. According to Hungarian regulations, wildlife management units are obliged to control the populations of non-protected mesocarnivore species, such as the European polecat (*Mustela putorius*), beech marten (*Martes foina*), European badger (*Meles meles*), red fox (*Vulpes vulpes*), and golden jackal (*C. aureus*). The legislative background of mesocarnivore population control is detailed on Zenodo (https://zenodo.org/records/15645626). As control and management activities are applied uniformly across the entire territory, the chance of a carnivore being harvested depends primarily on population distribution and the skills of the hunter. We hypothesised that this wildlife management practice ensured random sampling of the study populations. To ensure adequate detection power, the target sample size was set at 59 individuals per host species. This sample size is sufficient to detect a 5% prevalence at a 95% confidence level (Thrusfield and Christley, 2018).

For statistical analysis, the host species and sex, and the harvesting coordinates were recorded whenever possible (Fig. 1). For 46 red foxes (*V. vulpes*), 35 European badgers (*M. meles*), 9 golden jackals (*C. aureus*), and 6 stone martens (*M. foina*), sampling locations were recorded only at the hunting territory level, lacking precise coordinates. These specimens were included in the calculation of overall prevalence but were excluded from subsequent analyses.

### 2.3 Parasitological and molecular methods

The eyes of the carcasses were examined for nematodes within 72 h of collection. Both the conjunctival sacs and corneal surfaces were inspected, all recovered eyeworms were collected and preserved in 96% ethanol for morphometric and molecular analyses. Morphological identification was based on previous descriptions (Otranto et al., 2003b; Čabanová et al., 2018).

Given the ongoing expansion of *T. callipaeda* across the continent, all eyeworm specimens were hypothesised to belong to the same suprapopulation and thus, genetic lineage. To validate this assumption, a subsample of seven worms (1–2 per host) was selected for molecular analysis. These specimens originated from four infected animals (two golden jackals and two red foxes), representing both canid host species and two distinct study sites (Zselic Hills and Outer Somogy).

Genomic DNA was extracted from whole worms using the DNeasy Blood & Tissue Kit (Qiagen, Hilden, Germany) according to the manufacturer’s protocol. Nuclease-free water was used as the negative control. Partial fragments of the mitochondrial cytochrome *c* oxidase subunit 1 (*cox*1) gene were amplified using the specific primers NTF (5′-TGA TTG GTG GTT TTG GTA A-3′) and NTR (5′-ATA AGT ACG AGT ATC AAT ATC-3′) following Otranto et al. (2005b). For the PCR reaction, we used a Taq PCR core kit (Qiagen). PCR was performed using 25-μl reaction volumes, each containing 0.2 μl Taq DNA polymerase (Qiagen), 2.5 μl 10× buffer, 2 μl dNTPs (200 μM of each), 0.2 μl of each primer (50 μM), 1.0 μl template, 5 μl Q-solution and 15.4 μl nuclease-free water. The PCR conditions were as follows: initial denaturation at 95 °C for 5 min, followed by 35 cycles of denaturation at 94 °C for 30 s, annealing at 40 °C for 30 s, and extension at 72 °C for 1 min. The final extension was performed at 72 °C for 10 min, followed by a holding step at 12 °C. PCR products were separated by electrophoresis on 1.5% agarose gels and visualised under a GelDoc Go Gel Imaging System (Bio-Rad, Hercules, CA, USA) after staining with SYBRsafe (Invitrogen Thermo Scientific, Carlsbad, CA, USA). The sizes of the PCR products were estimated by comparison with a 100-bp DNA ladder (Invitrogen Thermo Scientific). The PCR products were purified using AMPure XP beads (Beckman Coulter Life Sciences, Indianapolis, IN, USA) and analysed using an ABI3730XL DNA analyser (Microsynth AG, Balgach, Switzerland) with the same PCR primers. The newly generated *cox*1 sequences were submitted to the GenBank database under the accession numbers PX498019-PX498025.

### 2.4 Phylogenetic analysis

Phylogenetic relationships among newly generated and retrieved from GenBank *cox*1 sequences for *Thelazia callipaeda* were inferred using the Maximum Likelihood (ML) method implemented in MEGA 12 (v. 12.0.11) (Tamura et al., 2024). Bootstrap support values were calculated based on 1000 pseudo-replicates. The newly generated sequences were compared with available data in GenBank using the BLASTn algorithm. Reference haplotype sequences from different geographical regions (see https://zenodo.org/records/18194284 File 05 for details and accession numbers) were retrieved from GenBank for phylogenetic analyses. The best-fitting evolutionary model (HKY+I; BIC = 4107.892) was estimated in MEGA 12 and subsequently applied in the ML analysis. Gaps and missing data were treated using partial deletion with a 95% site coverage cut-off, and all codon positions (1st, 2nd, and 3rd) were included. The initial tree was automatically generated using the Neighbor-Joining/BioNJ method, and the final ML tree was optimised using the Subtree-Pruning-Regrafting (SPR) algorithm with an extensive branch-swapping level.

### 2.5 Statistical analysis

#### 2.5.1 Dataset organisation

The investigation aimed to identify possible risk factors that could influence the occurrence of *T. callipaeda* infection in its wildlife reservoirs. Therefore, each carcass involved in the study was treated as a single observation and categorised as either infected or uninfected. Infected were defined as carcasses carrying eyeworms in the conjunctival sac. Carcasses without visible parasites in the conjunctival sac were classified as uninfected. These individual data points were linked to environmental variables through the geographical coordinates of the collection sites (see https://zenodo.org/records/18194284).

#### 2.5.2 Infection indices

Prevalence and mean intensity were calculated with their 95% confidence intervals (95% CI) for all harvested species. Since mustelids did not harbour the parasite, they were excluded from subsequent analyses. We assessed and compared the overall and species-level prevalence and mean intensity (defined as the average number of eyeworms per infected host) between canid hosts, as well as between sexes. These parameters were determined using the QPweb online application (https://www2.univet.hu/qpweb/qp10/index.php) (Reiczigel et al., 2019). The software computes 95% CIs for prevalence using Sterne’s method, while the 95% CIs for mean intensity were estimated using the bias-corrected and accelerated (BCa) method with 2000 bootstrap replications. Statistical comparisons of prevalence and mean intensity between groups were performed using Fisher’s exact test and a bootstrap 2-sample t-test with 1000 replications, respectively. Regarding the comparison of prevalence in subgroups, such as between sexes within a single host species, an unconditional exact test was applied.

#### 2.5.3 Geographical data collection and management

Red foxes and golden jackals lacking precise collection coordinates were excluded from spatial analysis. The involved canids were plotted on a map based on their coordinates and projected onto a 2.5 × 2.5 km UTM grid cell layer. This projection resulted in a total of 56 squares with detailed geographic, climate and vegetation data. Each square contained at least one animal. The UTM grid served as a spatial proxy for the habitat of the investigated host, forming the basis for further statistical calculations to determine the potential environmental drivers of *T. callipaeda* occurrence (Goodin et al., 2018).

To test the possible influence of human presence on *T. callipaeda* infection in carnivores, the distance of each sampling site from the nearest human settlement was calculated. To illustrate the spatial distribution of wild canids relative to human settlements, the collection coordinates were plotted in a diagram showing distances in 250-m intervals on the x-axis. In the statistical analysis, distance was used as an explanatory variable (DISTANCE).

#### 2.5.4 Determination of independent variables for statistical analysis

To determine the potential effects of environmental factors on thelaziosis in wild canids, geographical, climatic, land cover, and socio-economic data were collected for each UTM grid cell. The selection of potential environmental factors was based on prior evidence indicating their influence on the occurrence of *T. callipaeda* (Otranto et al., 2003a, 2006; Palfreyman et al., 2018). The proportion of different land cover categories, such as agricultural land (AGRO), forest cover (FOREST), grassland (GRASS) and the summarised area of wetland habitats and water bodies (WETHABIT) within UTM cells was calculated. As the most important intermediate host of *T. callipaeda* is *P. variegata* and this drosophilid species dominantly occurs in forested habitats (Otranto et al., 2006; Bernardini et al., 2024; González et al., 2025), forest typology of the UTM grid cells was also determined. Forest types that did not exceed 1% of the area were excluded. Employed variables were FAGUS (beech, *Fagus sylvatica*), QUCARP (oak species, *Quercus* spp., and hornbeam, *Carpinus betulus*, mixed forests), QUCERR (Turkey oak, *Quercus cerris*), PINUS (pine, *Pinus* spp. forests), ROBIN (black locust, *Robinia pseudoacacia*). Due to its patchy distribution within the study area, the presence or absence of beech was also included as a categorical variable (FAGUS_F) (Salamon-Albert et al., 2010; Fekete et al., 2014). The Ecosystem Map of Hungary (http://alapterkep.termeszetem.hu/) was used to classify the land cover types of the spatial units. The climatic characterisation of the UTM grid cells was based on the summer heat-moisture index (SHM). This index was calculated as SHM = MWMT/(MSP/1000), where MWMT is the mean temperature (°C) of the warmest month, and MSP is the precipitation sum in mm between May and September (Marchi et al., 2020). The lower the SHM, the cooler and more humid the climate tends to be. Climate data was obtained using the ClimatEU software (v. 4.63). Temperature and precipitation values were calculated based on data from the decade preceding 2020 (2011–2020) (Marchi et al., 2020). The ClimateEU software is available at https://sites.ualberta.ca/∼ahamann/data/climateeu.html (accessed 16 November 2025). The elevation of the central point of the UTM cell (ELEV) was determined by the application of https://www.advancedconverter.com/map-tools/find-altitude-by-coordinates (accessed 16 November 2025). To investigate the potential effect of the regional socio-economic status on the prevalence of *T. callipaeda* in wild carnivores, the District Development Index (DDI) was used. This index encompasses 21 different indicators of socio-economic development and was adopted from the 2019 report of the Hungarian Institute for Economic and Enterprise Research (GVI, 2019). Potential spatial effects were evaluated by treating the study area as a categorical variable (AREA) comprising three regions: Outer Somogy (OS), the Zselic Hills (ZS), and the Drava Plain (DP).

#### 2.5.5 Statistical analysis of candidate factors

**Fig. 2:**
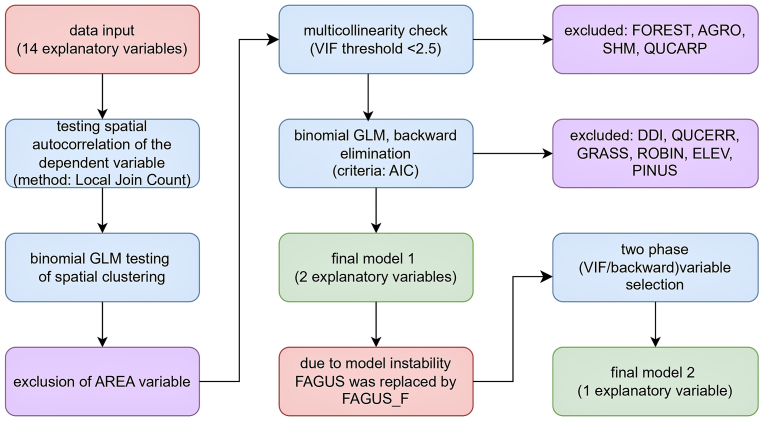
Flow chart of the variable selection and model building.

The statistical procedure aiming to determine the potential environmental factors of the maintenance of *T. callipaeda* within the study area is summarised in Fig. 2. Regarding the apparent geographical aggregation of the sample points, a preliminary test was conducted in GeoDa (v. 1.22) to assess spatial autocorrelation. Given the binary nature of the dependent variable (THCASE: infected/uninfected), the Local Join Count (LJC) statistics was employed, as it is specifically designed to identify spatial clusters in categorical data. The significance of spatial cores was determined through a conditional permutation test (9999 iterations) using Queen contiguity method for spatial weights. Locations were identified as significant cores where the spatial clustering of infected individuals (1-1 joins) significantly deviated from a spatially random distribution (*P* < 0.05) (Anselin and Li, 2019). To ensure robustness, in cases of LJC analysis yielded moderate significance (0.05 > *P* > 0.01), a generalised linear model (GLM) was subsequently conducted to verify the spatial autocorrelation.

All subsequent statistical analyses were performed within the R software environment (v. 4.4.2, 2024-10-31 ucrt) (R Core Team, 2024), with package *Rcmdr* (v. 2.9–5) (Fox and Bouchet-Valat, 2024). In the initial phase of variable selection, multicollinearity was assessed using variance inflation factors (VIF). Variables with the highest VIF values were removed manually in a step-by-step manner to prevent the loss of biologically relevant predictors (Sainani, 2013; Chowdhury and Turin, 2020; Xi et al., 2024). The manual variable selection process continued until all VIF values were less than 2.5, indicating tolerable multicollinearity among the remaining predictors (Johnston et al., 2018). The final models were obtained through backward stepwise selection based on Akaike’s information criterion (AIC) values. The discriminatory power of the model was assessed by receiver operating characteristic (ROC) curve analysis, specifically by calculating the area under the curve (AUC). For ROC curve analysis, R packages *pROC* (Robin et al., 2011), *ggplot2* (Wickham, 2016), and *verification* (Gilleland, 2025) were used. Our research data and the details of data processing are available at https://zenodo.org/records/18194284 with a README file adapted from Conzett and Dijkstra Haugstvedt (2024).

## 3 Results

**Fig. 3:**
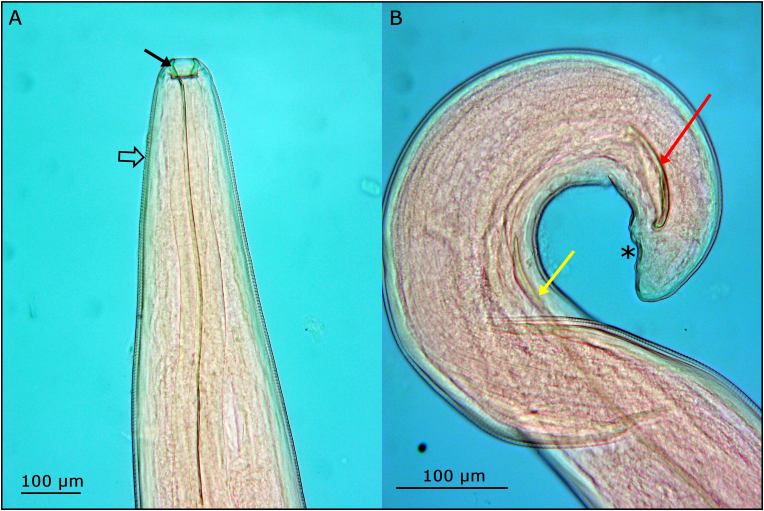
Morphological characteristics of an adult *Thelazia callipaeda* male. **A** Anterior region showing the hexagonal-shaped buccal cavity (*black arrow*) and cuticular striations on the anterior part (*empty arrow*). **B** Posterior region showing the shorter right spicule (*red arrow*), the longer left spicule (*yellow arrow*) and the cloacal papillae (*asterisk*).

**Fig. 4:**
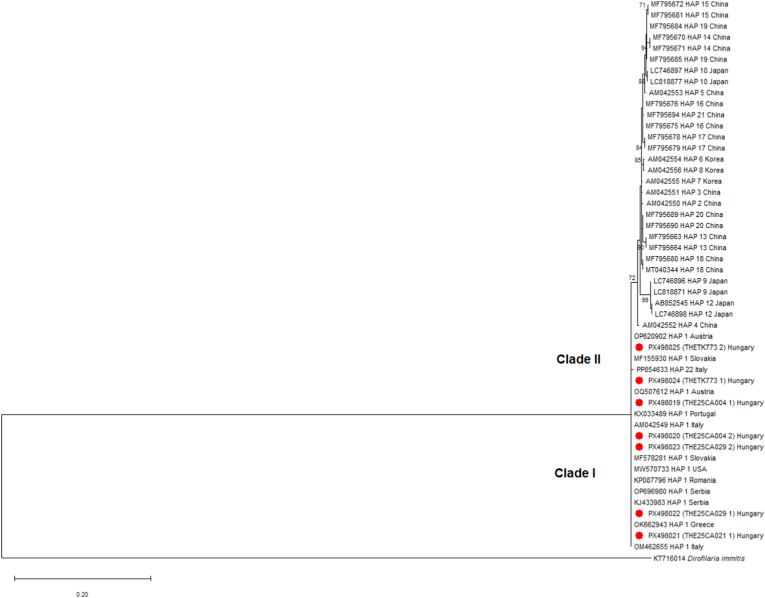
Maximum Likelihood consensus tree based on the Hasegawa-Kishino-Yano model of 51 nucleotide sequences of *Thelazia callipaeda* partial *cox*1 and the outgroup (*Dirofilaria immitis*). The two phylogenetic clades are indicated, and the newly generated sequences from canids in Hungary are marked with red dots. All positions with less than 95% site coverage were eliminated, i.e. fewer than 5% alignment gaps, missing data, and ambiguous bases were allowed at any position. Bootstrap support is indicated at the nodes; only values > 70% are shown.

All parasites isolated in the framework of our study were identified as *T. callipaeda* by morphological investigation (Fig. 3). Positive amplicons in PCR showed a band of *c.*700 bp in the agarose gel. All newly generated *cox*1 nucleotide sequences (*n* = 7) were translated into amino-acid sequences; there were no gaps or stop codons. The BLAST analysis of our sequences (GenBank: PX498019-PX498025) showed a 100% similarity to a sequence of *T. callipaed*a Haplotype h1 (GenBank: AM042549) (Otranto et al., 2005b). Sequence alignment (636 bp) of *cox*1 sequences retrieved from GenBank (including reference sequences for all 22 known haplotypes) revealed that the sequences for seven nematodes from two infected golden jackals and two infected red foxes belong to Haplotype 1 of *T. callipaeda*. In the ML phylogenetic consensus tree using the HKY+I model (Fig. 4), the present *T. callipaeda* isolates from golden jackals and red foxes all clustered together in Clade I (Haplotype 1) with good bootstrap support (74%). The remaining sequences retrieved from GenBank all originated from the Asian regions and were grouped within Clade II, represented by two moderately supported branches (Fig. 4). Overall, molecular identification confirmed that the parasites isolated from the local endemicity belonged to Haplotype 1 of *T. callipaeda*.

During this study, a total of 371 carnivores (red fox, *n* = 180; European badger, *n* = 119; golden jackal, *n* = 62; and stone marten, *n* = 10) were investigated. The predetermined sample size was achieved for canids and badgers, while the number of stone martens was insufficient for the reliable detection of the parasite. We found 28 infected animals, representing an overall prevalence of 7.5% (95% CI: 5.2–10.7%). The number of worms per infected host ranged from 1 to 15 specimens, with a mean intensity of 2.7 (95% CI: 1.9–4.0). Infection was restricted to foxes and jackals. The 242 wild canids showed an overall prevalence of 11.6% (95% CI: 8.0–16.3%). No statistically significant differences were observed between the two host species regarding prevalence (*P* = 0.82) or mean intensity (*P* = 0.81). In the red fox, prevalence and mean intensity were 12.2% (95% CI: 8.0–18.0%) and 2.6 (95% CI: 1.7–4.9), respectively; while in the golden jackal, these values were 9.7% (95% CI: 4.3–20.0%) and 3.0 (95% CI: 1.5–6.5). No sex-related differences in prevalence were detected, either overall or within species (Table 1). After excluding specimens lacking precise coordinates, 187 canids (20 infected and 167 uninfected) were included in the spatial analysis.

**Table 1:** Overall and within-species differences in prevalence and mean intensity of *Thelazia callipaeda* between the sexes of red fox and golden jackal. All animals were sampled in the South Transdanubian region of Hungary between November 2024 and July 2025.

	Prevalence (95% CI) (%)	Mean intensity (95% CI)	*P*-value
**Overall**			
Female (*n* = 116)	10.2 (5.8–16.9)	2.5 (1.6–4.2)	0.55
Male (*n* = 126)	12.9 (8.0–20.1)	2.88 (1.7–5.5)	0.76
**Red fox**			
Female (*n* = 86)	11.6 (6.2–20.2)	1.9 (1.3–2.6)	1
Male (*n* = 94)	12.8 (7.3–21.2)	3.3 (1.7–7.3)	0.34
**Golden jackal**			
Female (*n* = 30)	13.3 (4.7–29.8)	1.8 (1.0–2.5)	0.42
Male (*n* = 32)	6.2 (1.1–20.0)	5.5 (2.0–5.5)	–a

a There was too little variability in the data to calculate a bootstrap *P*-value.

**Fig. 5:**
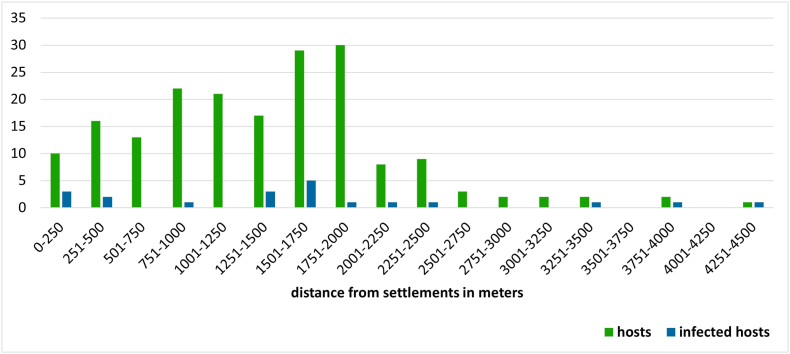
Distribution of animals harvested at various distances from settlements. For a finer assessment, distances were categorised into 250-m intervals. The columns represent the aggregate numbers of collected (*green*) and infected (*blue*) red foxes and golden jackals.

**Fig. 6:**
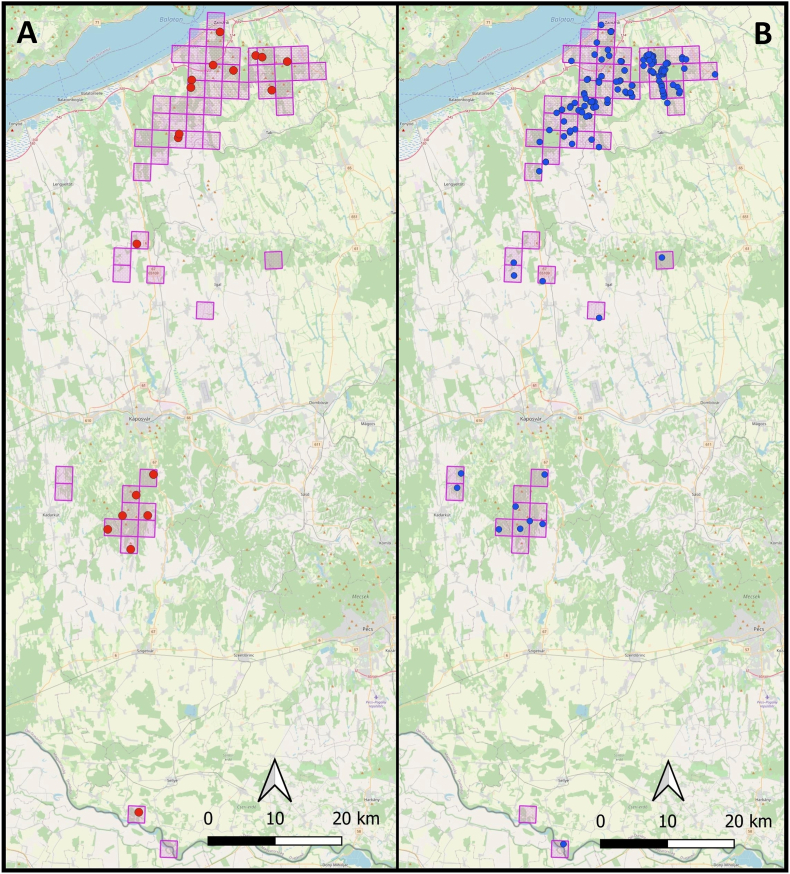
The collection sites of infected (**A**) and uninfected specimens (**B**) in 2.5 × 2.5 km UTM grids.

**Fig. 7:**
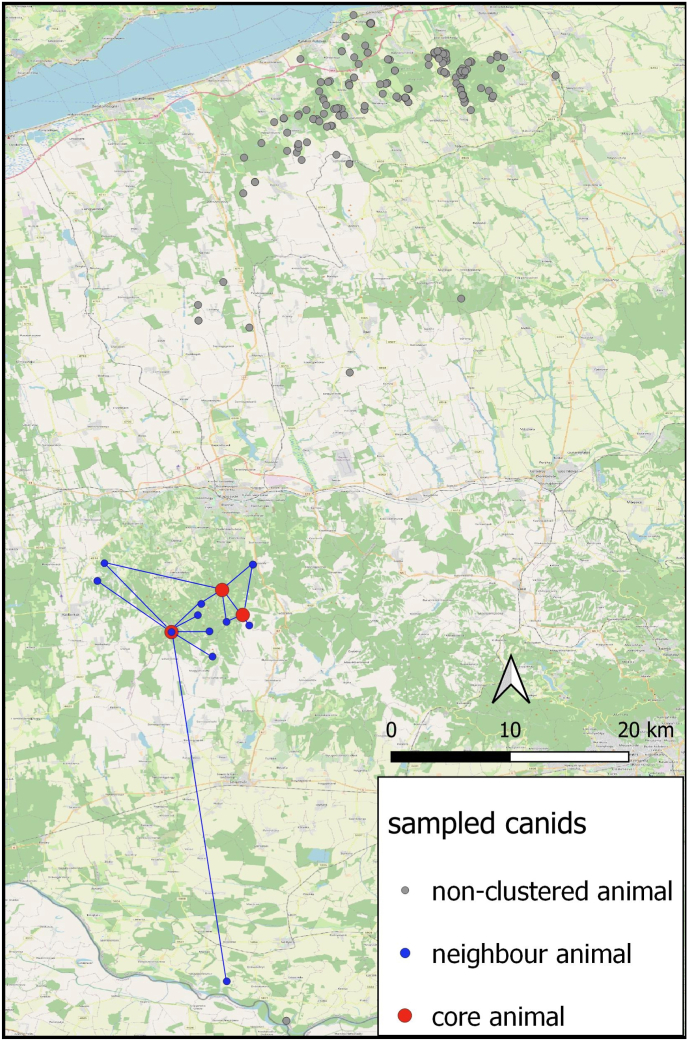
Spatial aggregation of positive animals confirmed by Local Join Count statistics in the Zselic region. *Note*: Blue lines indicate the connections between each core animal and its neighbours.

The majority (84.5%) of red foxes and golden jackals were hunted within 2000 m of human settlements (Fig. 5). After mapping the specimens’ positions (*n* = 187), we assigned them into 56 individual 2.5 × 2.5 km UTM grid cells (Fig. 6). Testing the spatial autocorrelation using LJC statistics revealed that three of the 187 animals, all within the Zselic region, had significantly more infected neighbours than expected by chance (*P* < 0.05). These individuals functioned as cluster cores, representing focal points of infection that encompassed both infected and uninfected neighbours (Fig. 7).

Despite these findings, the GLM did not confirm that the specific study areas contributed to the formation of spatial autocorrelation. Notably, neither the Outer Somogy nor the Zselic region had a significant effect on spatial clustering. The Drava Plain was excluded from this evaluation due to an insufficient sample size (*n* = 3) for a robust assessment (Table 2).

**Table 2:** Results of binomial GLM trained to evaluate the importance of areas.

	*β*	SE	*z*-value	*P*-value
Intercept	−0.6931	1.2247	−0.566	0.571
Outer Somogy	−1.9328	1.2639	−1.529	0.126
Zselic	0.2076	1.3046	0.159	0.874

*Abbreviation*: *β*, regression coefficient; SE, standard error.

In building the GLM, we initially found that the VIF exceeded its threshold (2.5) for four variables: FOREST (VIF = 38.33), AGRO (VIF = 6.98), SHM (VIF = 3.35), and QUCARP (VIF = 2.67). After addressing this issue using step-by-step removal, the VIF values of all remaining explanatory variables fell below the threshold. The initial model included nine explanatory variables; however, following backward selection, only two remained (FAGUS, DISTANCE). Consequently, the model’s AIC decreased from 126.38 to 118.51. Among the final variables, only FAGUS showed a significant effect. The multivariable logistic regression model supporting the role of beech forests as an independent risk factor for thelaziosis showed modest discriminatory power (AUC = 0.662), yet it performed significantly better (*P* < 0.001) than random classification. The second model, in which FAGUS was replaced by FAGUS_F was constructed using the same methodology. Following the initial VIF-based selection DDI, DISTANCE, ELEV, FAGUS_F, GRASS, PINUS, QUCERR, ROBIN and WETHABIT were identified as candidate variables for backward selection. As a result, the AIC decreased from 137.38 to 126.61, leaving FAGUS_F as the sole predictor. The discriminatory power of the model was lower (AUC = 0.60; *P* = 0.132) than the previous one; however, its stability was enhanced (Table 3).

**Table 3:** Results of the final binomial GLMs predicting the probability of *Thelazia callipaeda* infection.

	*β*	SE	*z*-value	*P*-value
First GLM				
intercept	−3.0253	0.5257	−5.755	<0.001
FAGUS	4660.00	31.300	694000.0	<0.001
DISTANCE	1.5100	0.8670	2.630	0.145

Second GLM				
intercept	−2.3979	0.2897	−8.278	<0.001
FAGUS_F	1.1658	0.5181	2.250	0.024

*Abbreviation*: *β*, regression coefficient; SE, standard error.

Regarding the impact of human activity, the beta coefficients for DDI and DISTANCE exhibited opposite signs in both models (DDI: −0.143 and −0.156; DISTANCE: 0.527 and 0.454), suggesting contrasting effects on the occurrence of the parasite. However, neither association was statistically significant (*P* > 0.05). Details of statistical analyses are available in the Zenodo repository (https://zenodo.org/records/18194284).

## 4 Discussion

We conducted an eco-epidemiological study in the South Transdanubian region of Hungary to identify possible drivers of *T. callipaeda* presence in wild carnivores. Since our study area was a highly forested, low-income region with diverse geomorphological characteristics and extensive natural habitats, it was considered suitable for analysing the specific impact of forest typology, microclimatic, and socio-economic conditions on *Thelazia* spp. infections in wild carnivores, which are known to serve as natural reservoirs.

Our findings regarding prevalence and mean intensity were consistent with those established in eastern Croatia, which borders the southern edge of our study area. The overall prevalence in canids in this study (11.6%) is consistent with reports from eastern Croatia (9.7–12.4%) (Gagović et al., 2025) and represents an intermediate level of infection. These values fall within the range defined by the lower prevalences reported in northern Italy (5.1%) (Otranto et al., 2003a) and Switzerland (5.6%) (Malacrida et al., 2008), and the markedly higher prevalences found in eastern Bosnia (50.0%) (Hodžić et al., 2014), and La Vera, Spain (61.3%) (Marino et al., 2018). The absence of infected mustelids (family Mustelidae) is consistent with the results observed in Croatia, where 180 European badgers (*M. meles*) and 215 stone martens (*M. foina*) were examined (Gagović et al., 2025). However, the susceptibility of mustelids has been evidenced in European badgers and stone martens in Romania (Ionică et al., 2019) as well as in sables (*Martes zibellina*) in the Russian Far East (Odoevskaya et al., 2015). It is suggested that the predetermined sample size was not sufficient to detect the low prevalence in the local mustelid populations. Haplotype 1 was identified in Hungary, and all neighbouring countries (Gajić et al., 2014; Hodžić et al., 2014; Mihalca et al., 2015; Gagović et al., 2025); this genetic uniformity likely resulted from the geographical spread of the infection.

The similarity in prevalence values between the directly connected homogeneous regions underlines the epidemiological relevance of common climatic and vegetation characteristics. The vast majority of Southern Transdanubia possesses a landscape structure comparable to that of eastern Croatia. However, the Zselic microregion differs in its topography and microclimatic characteristics, and consequently in its vegetation structure. This hilly area has a more humid and cooler microclimate than the surrounding landscape; therefore, its forest vegetation is dominated by oak-hornbeam (*Quercus petraea/C. betulus*) stands, with beech (*F. sylvatica*) occurring on northern slopes (Salamon-Albert et al., 2010). These unique microclimatic conditions are suggested to be the underlying reason for the frequent occurrence of *T. callipaeda* in the local carnivore population compared to other parts of our study area and eastern Croatia (Gagović et al., 2025).

On the other hand, the apparent infection accumulation in the Zselic region was only moderately supported by LJC analysis and was not confirmed as an independent spatial effect by the GLM. This suggests that the observed clustering was not an autonomous spatial phenomenon, but rather the result of the aggregation of underlying environmental drivers. These findings support our hypothesis that the central driver of thelaziosis in wild canids persists throughout Southern Transdanubia at varying intensities. Therefore, the cluster determined by LJC analysis represents a local peak in these regional ecological conditions.

As a result of backward stepwise selection, FAGUS (*P* < 0.001) and DISTANCE (*P* = 0.145) remained in the final model. Both variables exhibited disproportionately high beta coefficients, suggesting potential model instability. Despite this, the model showed only a modest discriminatory power (AUC = 0.66, *P* < 0.05), indicating that the identified environmental factor (extent of beech forests within the examined spatial unit) provides meaningful but partial insights into the occurrence of *T. callipaeda*. For this reason, a subsequent model was built using the occurrence of beech as a categorical variable (FAGUS_F). This model yielded a more stable and realistic beta coefficient; however, the ROC curve analysis indicated lower discriminatory power. Despite the reduction in discriminatory power, this latter model proved more robust than the previous one using the absolute proportion of beech forests as a variable. Although the conversion of a continuous variable to a binary one seems to result in loss of information, in this case, the presence or absence of a species with narrow ecological tolerance highlights the most interesting aspect of a phenomenon. The results support, although do not definitely prove, the relevance of humid and cool climatic conditions in maintaining thelaziosis within the study area. In this region, European beech (*F. sylvatica*) is the most hygrophilous tree species; consequently, its distribution is primarily determined by humidity levels in July (Führer et al., 2011), during the activity peak of *Phortica* flies (Otranto et al., 2006). In contrast, Turkey oak (*Q. cerris*) is the most xerotolerant native tree species in the Pannonian Floristic Province, and thus it forms almost pure stands within the driest parts of South Transdanubia. Consequently, the distribution of both hygrophilous and xerotolerant tree species correlates with the variation in SHM. This multicollinearity resulted in a high AIC, leading to the removal of SHM from the multivariable model. Pine and black locust stands showed no impact on thelaziosis in wild canids, likely due to their low representation in the study area.

Interestingly, neither the extent of forest cover nor elevation appeared to be relevant in the transmission of thelaziosis in the local wildlife. Regarding forest cover, it is likely that the highly forested nature of the overall landscape masks the well-known relevance of sylvatic habitats in the epidemiology of *T. callipaeda*. Regarding the elevation, although the study area possesses a very diverse topography, the altitudinal range between the lowest and the highest points was not sufficient to result in a significant difference in the occurrence of thelaziosis. The extent of agricultural land, grassland, surface waters, and wetland showed no association with the local *T. callipaeda* occurrence. These types of habitat served as a contrast to the forest cover. However, in the highly forested landscape, their effect was not apparent.

The risk-increasing impact of beech stands has not been investigated yet. Common features of different geographical regions with extended beech forests are worth investigating to determine the possible factors that contribute to the higher risk of eyeworm transmission. The *T. callipaeda* hyperendemic region of the Balkan Peninsula is mentioned to have extensive oak-beech mixed forests on 43% of the studied territory (Hodžić et al., 2014). Moreover, it is in one of the most important beech habitats of Europe as a former glacial refugium (Fuchs et al., 2024). Central China, where the incidence of human thelaziosis is the highest (Wang et al., 2014; Zhao et al., 2017), provided more glacial refugia for the genus *Fagus*, possessing the most diverse assembly (Cai et al., 2021). Besides higher relative humidity, beech forests provide a higher amount of deadwood than oak-dominated forests (Vandekerkhove et al., 2009). Although details of fruit flies’ ontogenesis have not been revealed, the potential relevance of decaying wood cannot be excluded as a supporting factor for intermediate host populations (Gornostaev et al., 2025). Research conducted in Hungary found that *Phortica* flies fed on tinder fungus (*Fomes fomentarius*) (Papp, 2002), the key fungal decomposer of beech forests (Dyson et al., 2024). However, the specific factors that directly impact the vectors warrant further investigation.

On the other hand, it should be noted that Turkey oak (*Q. cerris*) proved to be dominant in some European endemic areas (Otranto et al., 2006; Pombi et al., 2020; Bernardini et al., 2022), and beech species do not occur in the Russian Far East (Krestov, 2003; Okitsu, 2016). Therefore, it was assumed that not necessarily beech itself, but rather some environmental features related to beech forests may pose an increased infection risk. The investigation of ecological similarities of all known endemic areas of *T. callipaeda* might support a better appreciation of actual transmission drivers.

Our study area belongs to the South Transdanubian Region, which is one of the least developed administrative units of Hungary. Apart from the county seats and the districts along the shore of Lake Balaton, the majority of the area is characterised by socio-economic deprivation (Keresztes and Vámosi, 2008; Berkes and Dusek, 2023). This attribute of the region provided an opportunity to analyse the anthropogenic influence on wild carnivore thelaziosis. Consequently, distance to the nearest human settlement was employed as a proxy for human activity. Contrary to our preliminary hypothesis, the occurrence of infected animals was more frequent in natural habitats; however, this association was not significant. This finding did not support the assumption of a higher risk in synanthropic environments. Nevertheless, 84.5% of the carnivores were harvested within a 2000 m radius of human settlements. This latter finding is consistent with previous observations in the same territory (Moloi et al., 2025), reflecting the continent-wide trend of mesocarnivore populations occupying synanthropic habitats (Otranto et al., 2015; Ćirović et al., 2016).

On the other hand, regarding the District Development Index (DDI), the frequency of infected specimens appeared to be higher in districts with lower socio-economic status, suggesting that human activity might influence the risk of thelaziosis in wild canids. However, as this association was not statistically significant and the variable was automatically removed from the model, the apparent contradiction between distance and socio-economic status requires further investigation and spatial analysis to identify the underlying factors of higher epidemiological risk associated with district deprivation. Without surveillance in domestic dogs, the potential direction of disease transmission cannot be established. However, our findings regarding the higher frequency of infection in distal habitats support the assumption that *T. callipaeda* could be maintained in natural reservoirs even without a domestic cycle. The only autochthonous human case of *T. callipaeda* infection in Hungary to date supports our hypothesis concerning the dominance of the sylvatic reservoir, as the patient’s infection was linked to outdoor activity in the Bükk National Park within Hungary (Juhász et al., 2022). This area is dominated by relatively humid and cool beech forests (Garamszegi and Kern, 2014; Náfrádi and Sümegi, 2024), where most of the domestic dog cases in Hungary have been reported (Farkas et al., 2018).

Although our experiences regarding *T. callipaeda* appear to differ from previously established epidemiological patterns, this distinction is only apparent. Within the Mediterranean distribution area, Turkey oak (*Q. cerris*) has been identified as an indicator of *T. callipaeda* hotspots (Otranto et al., 2006; Pombi et al., 2020; Bernardini et al., 2022). Within our study area, Turkey oak characterises the driest zone due to its drought tolerance. However, Turkey oak-dominated forest stands also possess vertical stratification and adequate canopy cover, providing suitable microhabitats for the vector fruit flies, particularly in areas where higher altitude ensures sufficient rainfall for the development of a closed canopy. In the study area, Turkey oak forests appeared to play a less relevant role in the maintenance of *T. callipaeda* compared to more hygrophilous communities, such as beech and oak-hornbeam (*Q. petraea/C. betulus*) stands. Nevertheless, the parasite was still present within these Turkey oak forests.

This study found no significant difference in *T. callipaeda* infection levels between red foxes and golden jackals, as neither prevalence nor intensity showed significant variation. This suggests that both hosts carry equal importance in the local maintenance of the parasite. Furthermore, golden jackals have a remarkable migratory capacity (Spassov and Acosta-Pankov, 2019), positioning them as long-distance spreaders of *T. callipaeda*, similarly to wolves (Otranto et al., 2007; Nájera et al., 2020).

This study has certain limitations. As the investigation focused on the adult parasites within their natural habitat, data on intermediate hosts or potential definitive hosts within the domestic cycle were not involved. Consequently, this approach was not suitable for revealing the specific attributes of beech forests that contribute to the transmission of *T. callipaeda* between natural hosts. The balanced microclimate of these forest habitats likely influences the population density and activity of the vectors; however, biotic factors, such as food sources and breeding sites, might also support the maintenance of vector populations and, thus, the transmission success of *T. callipaeda*. Further dipterological investigations are required to ascertain the factors affecting fruit flies in beech forest environments. Furthermore, this study detected no infection in mustelids despite their susceptibility (Odoevskaya et al., 2015; Ionică et al., 2019). Although this result is consistent with studies from the Balkans (Gagović et al., 2025), a larger sample size would likely reveal the presence of the parasite in mustelids, as well. Finally, an apparent contradiction was observed between two levels of human impact: proximity to the nearest settlement and the socio-economic status of the district. The design and the statistical analysis of this study were not adequate to identify specific human activities that increase the risk of canine thelaziosis in districts with low socio-economic status. Without surveillance in domestic dog populations, the exact role of wild and domestic carnivores in the transmission cycle cannot be determined.

## 5 Conclusions

The study was conducted in an area characterised by a regional sub-continental climate with sub-Mediterranean influence, which contrasts with the typical hot and arid summers of central Hungary. These climatic attributes highlight the significance of sustained summer moisture for the maintenance of *T. callipaeda* near its Mediterranean range. Hygrophilous forest stands, such as beech (*F. sylvatica*) forests, were detected to be proxies of habitat suitability for *T. callipaeda*. Although the results suggested the reservoir role of wild canids in the parasite’s transmission cycle, the risk-increasing effect of uncared-for domestic dogs could not be excluded, as wild canids in less developed districts showed a higher frequency of infection. To gain a clearer understanding of the dynamics of the spread of *T. callipaeda* in Central and Eastern Europe, it would be beneficial to study areas with various characteristics, such as continental dry zones, montane humid climate belts, and districts with different socio-economic status in the Carpathian Basin.

## Ethical approval

All animals used in the study were taken in legal hunting activities within the framework of the national game management programme. None of the animals were killed for the purpose of this study. The animals were shot in accordance with the Wildlife Management Act (Act LV of 1996) and its implementing regulation (Ministry of Agriculture and Rural Development Regulation 79/2004), which regulates the Hungarian wildlife management programme. The authors also declare that no live animals were used in the research and no experiments were conducted. More details can be found at https://doi.org/10.5281/zenodo.15645626.

## CRediT authorship contribution statement

**Eszter Nagy:** Conceptualization, Investigation, Data curation. **Rebeka Ráhel Nagy:** Investigation, Data curation. **Máté Miklós:** Investigation, Data curation, Visualization. **Sándor Szekeres:** Investigation. **Bawan Mustafa Abdalrahman:** Investigation. **Gábor Földvári:** Conceptualization, Writing – review & editing. **Lajos Rózsa:** Conceptualization, Methodology, Writing – review & editing. **Éva Fok:** Writing – review & editing. **Tamás Sréter:** Conceptualization, Writing – review & editing. **Tamás Tari:** Investigation, Data curation. **Melinda Kovács:** Writing – review & editing, Supervision. **Ágnes Csivincsik:** Conceptualization, Methodology, Data curation, Investigation, Writing – original draft, Writing – review & editing. **Gábor Nagy:** Conceptualization, Data curation, Visualization, Investigation, Writing – original draft, Writing – review & editing.

## Statement on the use of AI-assisted technologies

We used Grammarly (https://www.grammarly.com/) to correct grammatical errors and improve readability. All content was then reviewed and edited. The authors take full responsibility for the content of the published article.

## Funding

This research was supported by the National Research, Development and Innovation Office in Hungary (RRF-2.3.1-21-2022-00006). The work of Eszter Nagy was supported by the EKÖP-24-3-II programme of the National Research, Development and Innovation Fund (grant number: 2024-2.1.1-EKÖP-2024-00007). The contributions of Ágnes Csivincsik, Bawan Mustafa Abdalrahman, Melinda Kovács, and Gábor Nagy were supported by the Flagship Research Groups Programme of the Hungarian University of Agriculture and Life Sciences. Lajos Rózsa was also supported by the Slovak Research and Development Agency under contract APVV-22-0440. Sándor Szekeres was supported by the STARTING 150266 project, has been implemented with the support provided by the Ministry of Culture and Innovation of Hungary from the National Research, Development and Innovation Fund, financed under the STARTING_24 funding scheme.

## Declaration of competing interests

The authors declare that they have no known competing financial interests or personal relationships that could have appeared to influence the work reported in this paper.

## Data Availability

The newly generated sequences have been uploaded to the GenBank database under the accession numbers PX498019-PX498025. All data generated or analysed during this study are openly available in the Zenodo repository at https://zenodo.org/records/18194284.
